# Coincidence analysis: a new method for causal inference in implementation science

**DOI:** 10.1186/s13012-020-01070-3

**Published:** 2020-12-11

**Authors:** Rebecca Garr Whitaker, Nina Sperber, Michael Baumgartner, Alrik Thiem, Deborah Cragun, Laura Damschroder, Edward J. Miech, Alecia Slade, Sarah Birken

**Affiliations:** 1 Duke-Margolis Center for Health Policy, 100 Fuqua Drive, Box 90120, Durham, NC 27708 USA; 2 Duke University School of Medicine, Department of Population Health Sciences, 215 Morris Street, Durham, NC 27701 USA; 3 University of Bergen, Department of Philosophy, Postboks 7805, 5020 Bergen, Norway; 4 University of Lucerne, Frohburgstrasse 3, P.O. Box 4466, 6002 Lucerne, Switzerland; 5 Department of Global Health, College of Public Health, University of South Florida, 3802 Spectrum Boulevard, Tampa, FL 33612 USA; 6 VA Ann Arbor Center for Clinical Management Research, University of Michigan, North Campus Research Complex, 2800 Plymouth Road, Building 18, Ann Arbor, MI 48109-2800 USA; 7 Center for Health Services Research, Regenstrief Institute, 1101 West 10th Street, Indianapolis, IN 46202 USA; 8 Avalere Health, 1201 New York Avenue NW, Suite 1000, Washington, DC 20005 USA; 9 Department of Implementation Science, Wake Forest School of Medicine, 525@Vine Room 5219, Medical Center Boulevard, Winston-Salem, NC 27157 USA

**Keywords:** Coincidence analysis, Configurational comparative methods, Causal inference, Comparative analysis

## Abstract

**Background:**

Implementation of multifaceted interventions typically involves many diverse elements working together in interrelated ways, including intervention components, implementation strategies, and features of local context. Given this real-world complexity, implementation researchers may be interested in a new mathematical, cross-case method called Coincidence Analysis (CNA) that has been designed explicitly to support causal inference, answer research questions about combinations of conditions that are minimally necessary or sufficient for an outcome, and identify the possible presence of multiple causal paths to an outcome. CNA can be applied as a standalone method or in conjunction with other approaches and can reveal new empirical findings related to implementation that might otherwise have gone undetected.

**Methods:**

We applied CNA to a publicly available dataset from Sweden with county-level data on human papillomavirus (HPV) vaccination campaigns and vaccination uptake in 2012 and 2014 and then compared CNA results to the published regression findings.

**Results:**

The original regression analysis found vaccination uptake was positively associated only with the availability of vaccines in schools. CNA produced different findings and uncovered an additional solution path: high vaccination rates were achieved by either (1) offering the vaccine in all schools or (2) a combination of offering the vaccine in some schools and media coverage.

**Conclusions:**

CNA offers a new comparative approach for researchers seeking to understand how implementation conditions work together and link to outcomes.

Contributions to the literature

- Coincidence Analysis (CNA) represents a new mathematical, cross-case method for researchers evaluating the implementation of complex interventions in dynamic settings.
- CNA can address multiple dimensions of real-world complexity, including conjunctivity (where several conditions must be jointly present to bring about an outcome) and equifinality (where different paths can lead to the same outcome). CNA can also detect causal chains, where conditions lead to an intermediary outcome, which then leads to the final outcome.
- Intentionally designed to investigate different hypotheses and uncover different properties of causal structures than more traditional approaches, CNA can identify implementation-related findings that might otherwise go undetected.

## Background

One of the basic analytic challenges within implementation science is to study and understand implementation within real-world, dynamic settings. Implementation of multifaceted interventions typically involves many diverse elements working together in interrelated ways, including intervention components, implementation strategies, and features of local context. Furthermore, boundaries between an intervention, its implementation, and its contextual features can prove difficult to discern in practice [1, 2].

For researchers seeking to explain these complex relationships encountered in real-world settings, causal inference can play an important role. Since the mid-1980s, Configurational Comparative Methods (CCMs) have increasingly been recognized as effective methods for causal inference, especially in the social sciences. The cumulative number of CCM-related publications listed in the core collection of the Web of Science [3] has dramatically escalated in recent years, with more total publications appearing during the 3-year period from 2017 to 2019 than in the entire preceding 22-year period between 1995 and 2016.

CCMs have also started to make prominent appearances within the health services research and implementation science literatures. CCMs, for example, were used in a recent Cochrane Review to identify conditions directly linked with successful implementation of school-based interventions for asthma self-management [4], featured as an innovative member of the mixed-methods repertoire in a major methodological review in public health [5], highlighted as a central method in newly published implementation science protocols [6, 7], and applied to determine different pathways for federally qualified health centers to achieve patient-centered medical home status [8]. CCMs were also featured in a dedicated chapter in the 2020 *Handbook on Implementation Science* [9].

CCMs are designed to investigate different hypotheses and uncover different properties of causal structures than traditional regression analytical methods (RAMs) [10, 11]. Qualitative Comparative Analysis (QCA) is one kind of CCM that, to date, has been most frequently applied in implementation science and health services research. The purpose of this article is to introduce a new CCM to the implementation research community: Coincidence Analysis. Coincidence Analysis (CNA) is a mathematical, cross-case approach that can be applied as a standalone method or in conjunction with other methods (including RAMs) to support causal inference and is available via the R-package cna [12–14].

CNA offers a new cross-case method for implementation and health services researchers exploring causality when evaluating or implementing multifaceted interventions in complex contexts. Investigators applying CNA can conduct analyses across entire datasets to identify specific combinations of components and conditions that consistently lead to outcomes and can be applied to large-*n* as well as small-*n* studies. Peer-reviewed, implementation-related work specifically involving CNA has started to emerge in implementation science, including podium presentations at major implementation conferences [15, 16], methods workshops dedicated specifically to CNA [17], published protocols [18], and full-length articles in established journals [19–23].

CNA is a new comparative approach that can be used by the implementation research community to support causal inference, answer research questions about conditions that are minimally necessary or sufficient, and identify multiple causal paths to an outcome. We present this article in three parts. In part 1, we establish the theoretical foundation for CCMs, define CNA as a method within the CCM family, and describe what CNA (and CCMs) uniquely offer. In part 2, we illustrate CNA by applying the method to a publicly available dataset that was originally analyzed using RAMs. In part 3, we offer guidance for reporting CNA design and results, and we discuss the limitations and challenges of CNA. In the additional files accompanying this article, we provide detailed descriptions of the steps and coding used to conduct the analysis (see Additional file 1) and the analytic dataset used (see Additional file 2) along with the R script (see Additional file 3) to allow for independent replication and validation of results.

## Part 1: laying the theoretical foundation for CCMs

### Defining causal inference in CCMs

CNA is one method within a class of CCMs used to model complex patterns of conditions hypothesized to contribute to an outcome within a set of data. CCMs search for causal relations as defined by a regularity theory of causality, according to which a cause is a “difference-maker” of its effect within a fixed set of background conditions. More specifically, *X* is a cause of *Y* if there exists a fixed configuration of background factors *Φ* such that, in *Φ*, a change in the value of *X* is systematically associated with a change in *Y*. If *X* does not make a difference to *Y* in any *Φ*, *X* is redundant to account for *Y* and, thus, not a cause of *Y*. The most influential theory defining causation along these lines is Mackie’s INUS-theory [24], with refinements by Graßhoff and May [25] and Baumgartner [26]. An INUS condition of an outcome *Y* is an insufficient but necessary part of a condition that is itself unnecessary but sufficient for *Y*. To use a common example for illustrating INUS conditions: not every fire is caused by a short circuit—fires can also be started by, for example, arson or lightning. However, a short circuit in combination with other conditions—e.g., presence of flammable material and absence of a suitably placed sprinkler—is sufficient for a fire. In this example, the short circuit is an INUS condition: it is a necessary, but itself insufficient, part of a sufficient, but itself unnecessary, condition for a fire. This particular causal path to a fire includes the combination of three specific conditions: presence of a short circuit, presence of flammable material, and absence of a sprinkler. All three of these conditions are difference-makers, for if one of them is missing, the fire does not occur along this causal path.

Regularity theories account for the Boolean properties of causation, which encompass three dimensions of complexity. The first is conjunctivity: to bring about an outcome, several conditions must be jointly present. For example, in a study of high-performance work practices and frontline health care worker outcomes, Chuang and colleagues [27] found that no single high-performance work practice was alone sufficient to produce the outcome of high job satisfaction. Instead, a configuration consisting of creative input, supervisor support, and team-based work practices together accounted for 65% of highly satisfied frontline health care workers [27]. Chuang and colleagues identified a second configuration that also led to high job satisfaction: supervisor support, incentive pay, team-based work, and flexible work [27]. Both configurations resulted in high job satisfaction independently of each other. These configurations illustrate equifinality, a second dimension of complexity where different paths can lead to the same outcome. The third dimension of complexity is sequentiality: outcomes tend to produce further outcomes, propagating causal influence along causal chains. For instance, high job satisfaction of health care workers may, in turn, promote patient satisfaction [28].

Why use CCMs in implementation research? CCMs study different properties of causal structures than RAMs and thus are appropriate for exploring different types of hypotheses. RAMs examine statistical properties characterized by probabilistic or interventionist theories of causation. In the probabilistic framework, *X* is a cause of *Y* if, and only if, the probability of *Y* given *X* is greater than the probability of *Y* alone and there does not exist a further factor, *Z*, that explains (i.e., neutralizes) the probabilistic dependence between *X* and *Y* [29, 30]. In the interventionist framework, *X* causes *Y* if, and only if, there exists an intervention on *X* that changes the outcome *Y* while causes on other paths to *Y* are fixed. The interventionist theory of causation is counterfactual in that a case cannot simultaneously “receive” and “not receive” an intervention; instead, the intervention model maps possible values of *Y* onto possible values of *X*, focuses on how variables *X* and *Y* relate to one another, and generates average treatment effects over a population [11, 31].

Conversely, CCMs trace Boolean properties of causal structures as described by regularity theories of causation, according to which *X* is a cause of *Y* if, and only if, X is an INUS condition of *Y* (see INUS definition above) [11, 24]. CCMs study implication hypotheses that link specific values of variables as “X = χ_i_ is (non-redundantly) sufficient/necessary for Y = γ_i_” [11, 14]. In this way, CCMs, including CNA, model the effect of conditions (e.g., high degree of *X*) on outcomes. This is a fundamentally different vantage point than the one adopted by RAMs which examine covariation hypotheses that link variables. Further, CCMs are case-oriented methods, in which observations consist of bounded, complex entities (e.g., organizations) that are considered as a whole [32]. A case-based unit of analysis differs from the approach taken in RAMs, where cases are deconstructed into a series of variables, and estimates represent the net effect of a variable for the average case. As CNA and other CCMs employ a case-based approach and thus can be used to identify which interventions work in an array of contexts, they present opportunities for implementation and health services research questions in particular.

### Different types of CCMs

While CCMs have a common regularity theoretic foundation, different CCMs rely on different a priori conceptions of outcome and causal factors and build causal models in different ways. For example, Qualitative Comparative Analysis (QCA), in its standard implementation that uses the Quine-McCluskey (QMC) algorithm [33, 34] requires identification of exactly one factor as outcome. It begins by identifying maximal sufficient and necessary conditions of the outcome, which are subsequently minimized using standard inference rules from Boolean algebra to arrive at a redundancy-free solution composed of INUS conditions of the outcome [10]. However, the QMC algorithm was not originally designed for causal inference. One consequence is that the non-observation of cases instantiating empirically possible configurations of the analyzed factors, also known as limited diversity, forces QMC to draw on counterfactual reasoning that goes beyond available data, sometimes requires assumptions contradicting the very causal structures under investigation [35], and regularly fails to completely eliminate redundancies in the presence of noise [14]. Moreover, QMC has built-in protocols for ambiguity reduction when multiple models fit the data equally well. Viable models are often eliminated to reduce ambiguity without justification, which is problematic for causal discovery [36].

### Advantages of using CNA

Coincidence Analysis (CNA) is a new addition to the family of CCMs [37, 38]. It uses an algorithm specifically designed for causal inference, thus avoiding the problems mentioned above. In particular, it does not build causal models by means of a top-down approach that first searches for maximal sufficient and necessary conditions and then gradually minimizes them using the QMC algorithm. Rather, CNA employs a bottom-up approach that first tests single factor values for sufficiency and necessity, and then tests combinations of two, three, etc. [13, 14]. All sufficient and necessary conditions revealed by this approach are automatically minimal and redundancy-free.

Additionally, CNA is designed to treat any number of factors as endogenous and is therefore capable of analyzing causal chains, or common-cause structures [39]. For example, Baumgartner and Epple (2014) found in a Swiss policy analysis that certain population, economic, or political characteristics in some areas led to a higher rate of prejudice and, in turn, discriminatory policy [39]. Analyzing causal chains may be advantageous if, for example, an intervention A occurs as a result of other factors but is not the ultimate outcome of interest. Identifying the full causal model, including which factors produce A on the path to the ultimate outcome of interest, is valuable when seeking to understand causal complexity. CNA is the only member of the CCM family that builds and evaluates models representing causal chains.

## Part 2: demonstrating CNA using publicly available data

### Data source

In March 2016, Rehn and colleagues reported the impact of implementation strategies on human papillomavirus (HPV) “catch-up vaccination” uptake in Sweden among fifth- and sixth-grade girls [40]. The purpose of the original study was to estimate the impact of various information channels and delivery settings on county-level catch-up vaccine uptake to inform future vaccination campaigns in Sweden.

The authors obtained county-level data on catch-up vaccinations and the eligible population from administrative data. They collected implementation strategies from county health care offices via an open-ended questionnaire emailed in 2012 asking respondents to list and describe “information channels” used to reach eligible girls and the settings in which they offered the vaccine. A subsequent phone interview was conducted in 2014 to update the lists.

Rehn and colleagues used regression analysis to estimate county-level catch-up vaccine uptake as a function of information channels and delivery settings. The authors concluded that the availability of vaccines in schools explained differences in county-level vaccine uptake; no information channels were found to make a difference in uptake.

Rehn and colleagues defined the outcome and predictor variables as follows:

*Outcome variable*: County-level catch-up vaccine uptake was defined as the percent of eligible girls born between 1993 and 1998 who received at least one dose of vaccine by 2014.

*Predictor variables*: Ten variables represented information channels and four variables represented the delivery settings where the vaccinations were available (some schools, all schools, primary health care centers, and other health care centers). All 14 factors were dichotomized with values of 1 (present) or 0 (absent).

All county-level data on vaccine uptake, information channels, and delivery settings used for the CNA illustration were reported in the article.

## Methods

We re-analyzed the data using CNA. A step-by-step guide for conducting CNA, using this study as an illustration, is provided in a document accompanying this article (see Additional file 1) as well as the analytic dataset (see Additional file 2) and the R script (see Additional file 3) used in the analysis.

### Step 1: define, calibrate, and select the factors (i.e., outcomes and conditions) to create a data set

Vaccination rate represented the outcome of interest and ranged from 49 to 84% across the 21 counties. We selected 65% as the threshold defining “high” catch-up vaccination rates after conducting sensitivity analyses in which we varied the threshold for “high-uptake,” using two different existing break points in the data: the 65% cut-off generated the greatest diversity among cases for the conditions and the outcome and yielded a sufficiently high number of cases featuring the outcome. We coded the 21 counties into a new dichotomous outcome called HI_UPTAKE where 1 = “catch-up vaccination rate of 65% or higher” and 0 = “catch-up vaccination rate less than 65%” (see Additional file 1 for details on the rationale for each step in the analytic process). A secondary analysis identified conditions leading to the absence of the outcome (HI_UPTAKE = 0) because conditions that prevent the outcome may differ from those that contribute to the presence of the outcome.

We prepared a dataset that included the uptake rates, delivery settings, and implementation strategies as reported by Rehn and colleagues (see Additional file 2 to view the analytic dataset used in the Coincidence Analysis). We transformed a number of factors from the original dataset for use with CNA because in the original dataset these factors had characteristics unsuitable for CCM processing. For instance, the original data set contained the factor “Primary health care centre” (PHC) that was constantly present in all 21 counties (cases). Constant factors like PHC can be automatically excluded as difference-makers. Another delivery setting, “Other health care center” (HC) was eliminated given limited variation across cases. We combined “All schools” (“organized delivery of the vaccine in all schools in the county;” AS = 1) and “Some schools” (“organized delivery of the vaccine in schools in some of the municipalities in the county…;” SS = 1) into a new multi-value ordinal factor called “SCHOOLS,” where SCHOOLS = 0 if AS = 0 and SS = 0; SCHOOLS = 1 if SS = 1 and AS = 0; and SCHOOLS = 2 if AS = 1. The resulting dataset included 12 potential explanatory factors. These factors could be combined into 6144 logically possible configurations, which could not be covered to an informative degree by the 21 cases included. Thus, the diversity index for the original data, i.e., the ratio of observed configurations to all possible configurations, was exceedingly small. The smaller the diversity index, the more challenging it is to draw informative configurational conclusions. To improve the diversity index, we included a subset of the 12 exogenous factors in our analysis. This is analogous to maximizing degrees of freedom in RAM.

We selected schools (SCHOOLS) and four of the ten information channels to include school-based information (SBI), media coverage (MC), social media (SM), and Cinema commercial/YouTube (CCY). Our rationale for choosing these four implementation strategies was that they were directly linked to school and digital media, two immersive domains that are dynamic and interactive where students and their parents commonly encounter new information, and thus likely to be effective channels for conveying information about why, where, how, and when to access vaccinations

In our initial analysis plan, three of the seven cases (counties) exhibiting the outcome (high uptake) instantiated exactly the same configuration of conditions, leaving only five observed configurations featuring HI_UPTAKE = 1 out of a total of 48 logically possible configurations. As we could not justify removing one of the four selected information channels on a theoretical basis alone, but still wanted to decrease the number of overall factors in the analytic dataset (and thus increase the diversity index), we decided to assess if we could combine two of the information channels into a single “meta-factor”—a common approach in CCMs to reduce the number of conditions without eliminating either of the properties represented by these conditions from the analysis. There are six possible ways to pair four different information channels. Accordingly, we created six different datasets (i.e., analytic samples), each representing a different pairing of two channels, the two remaining channels and the outcome HI_UPTAKE. We coded the new meta-factor in each analytic sample with the value 1 if, and only if, at least one of the two aggregated channels was present in a county (to view these six datasets, see Additional file 2).

### Step 2: perform CNA using the cna package in R [13]

Two parameters of fit—consistency and coverage—provide insight into the strength of the dependence between conditions and the outcome. Consistency, with a score ranging from 0 to 1, measures the degree to which the cases that instantiate a configuration or a whole model also instantiate the outcome [10]. Low-consistency values indicate that the dependence between conditions or models and the outcome is far away from a strict Boolean (deterministic) dependence. Coverage scores range from 0 to 1 and represent the proportion of cases with the outcome that also instantiate a particular configuration or whole model. Coverage measures a given configuration’s or model’s empirical importance based on the available data [10]. When applying CNA to the dataset, we set our minimum consistency and coverage scores to 1.0 [10]. However, it is important to note that CNA, and CCMs generally, risk overfitting model solutions when searching for maximal consistency and coverage thresholds, which can lead to false positives [41]. For this reason, consistency and coverage thresholds should only be set to 1 if researchers have strong reasons to assume that the data quality is very high (i.e., low levels of noise and measurement error); if that cannot be assumed, then researchers can apply a search strategy that systematically varies consistency and coverage thresholds to measure fit-robustness, or the degree to which a model solution agrees with other models identified at different consistency and coverage thresholds in the same dataset [42].

## Results

### Step 3: interpret results and refine model inputs if necessary

Like QCA, CNA encourages an iterative approach where researchers can run analyses, interpret results, and redefine model inputs before finalizing a model set [32]. As such, we discuss our interpretation of the findings as our iterative analyses progress.

Our analyses produced model ambiguity, meaning that the data were insufficient to determine exactly which causal structure was operative. Of the six analytic samples, three datasets yielded a total of five causal models for HI_UPTAKE = 1, all of which featured maximal consistency and coverage scores (see the R replication script for a complete list of models). All models had the following identical terms as part of their solution, where “+” symbolizes the Boolean operator OR, “*” symbolizes AND, and “↔” expresses sufficiency and necessity:

1 $$ \mathrm{SCHOOLS}=2+\mathrm{SCHOOLS}=1\ast \mathrm{MC}=1\leftrightarrow \mathrm{HI}\_\mathrm{UPTAKE}=1 $$

The above expression (1) translates to “counties had high catch-up vaccination rates if, and only if they offered vaccination in all schools OR offered vaccination in some (but not all) schools AND used a media coverage implementation strategy.” All five causal models resulting from our analysis were supersets of (1), so we concluded that the factor values contained in (1) were causally relevant for high uptake. However, (1) was not a complete model because it only achieved a consistency score of 0.875 (i.e., only 87.5% of counties with this configuration were high-uptake counties). Expression (1) covers a county, Skåne, that exhibited the configuration SCHOOLS = 1 * MC = 1 but was not a high uptake. The other counties with this configuration, SCHOOLS = 1 * MC = 1, were always associated with high uptake of vaccines. Thus, other factors unique to Skåne must be missing from expression (1).

While our data were insufficient to identify the factors that distinguished Skåne from other counties with the same configuration, the five complete models inferred by CNA provided five possible explanations. Interestingly, all five of these solution models combined SCHOOLS = 1 * MC = 1 with the absence of other information channels. Only when SCHOOLS = 1 * MC = 1 was accompanied by SM = 0 or SBI = 0 or CCY = 0 was it associated with high uptake with 100% consistency, which could indicate these other information channels produced a “backfiring” effect.

**Fig. 1:**
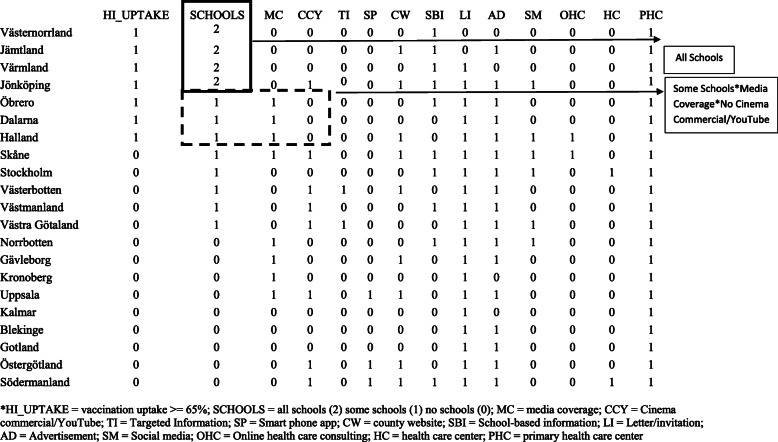
Overall model identified by Coincidence Analysis with 100% consistency and 100% coverage. *HI_UPTAKE = vaccination uptake > = 65%; SCHOOLS = all schools (2) some schools (1) no schools (0). *MC* media coverage, *CCY* cinema commercial/YouTube, *TI* targeted information, *SP* smart phone app, *CW* county website, *SBI* school-based information, *LI* letter/invitation, *AD* advertisement, *SM* social media, *OHC* online health care consulting, *HC* health care center, *PHC* primary health care center

After further reviewing data for each county, we determined that the most plausible implementation strategy to backfire was cinema commercials and/or YouTube. Online media like YouTube can backfire because these open-source platforms contain unvalidated content that can appear automatically through newsfeeds or advertisements, potentially overriding legitimate health-related information [43]. Furthermore, we observed a negative relationship between the presence of CCY and the vaccination rate outcome in the underlying dataset. As Fig. 1 shows, the presence of CCY was relatively well-represented in the overall dataset: 8 of the 21 cases had CCY = 1. In 7 of these 8 cases, HI_UPTAKE = 0. The lone exception was the county of Jonkoping, where the sufficient condition of SCHOOLS = 2 was also present. For these reasons, we deemed the following complete model to be the most plausible:

2 $$ \mathrm{SCHOOLS}=2+\mathrm{SCHOOLS}=1\ast \mathrm{MC}=1\ast \mathrm{CCY}=0\leftrightarrow \mathrm{HI}\_\mathrm{UPTAKE}=1 $$

Expression (2) translates to “counties had high catch-up vaccination rates if, and only if they offered vaccination in all schools OR offered vaccination in some (but not all) schools AND used a media coverage implementation strategy but not cinema commercials/YouTube.” Expression (2) had perfect consistency and coverage scores (1.0, respectively) and differentiated Skåne (a county without high uptake) from high-uptake counties that also had vaccinations available at some schools and used media coverage. Figure 1 highlights the configurations instantiating this model in the data.

Applying the same analytic approach to model the absence of the outcome yielded seven models for HI_UPTAKE = 0 such that six of these seven models had a common core that corresponded exactly to the negation of the core of the positive models:

3 $$ \mathrm{SCHOOLS}=0+\mathrm{SCHOOLS}=1\ast \mathrm{MC}=0\leftrightarrow \mathrm{HI}\_\mathrm{UPTAKE}=0 $$

Expression (3) translates to “counties had no high catch-up vaccination rates if, and only if they did not offer vaccination in all schools OR offered vaccination in some (but not all) schools AND did not use a media coverage implementation strategy.” Expression 3 exhibited 1.0 consistency and 0.93 coverage. Taken together, these results provide substantive evidence that media coverage is relevant for differentiating counties with and without high vaccination uptake, adding additional information to the regression results of the original study.

## Discussion

The results of this CNA indicate that, under specific conditions, information channels made a difference for high vaccination uptake. This contrasts with the results of the regression analysis from the original study, which concluded that information channels made no difference in increasing vaccination uptake. Our results imply that the availability of vaccination in some schools is only sufficient for high vaccination rates if media coverage is employed and certain other communication channels are not used. In other words, the data contain enough evidence to infer that when vaccination is available at some but not all schools, availability must be complemented by media coverage to achieve high uptake. The data do not contain enough evidence, however, to have absolute certainty which communication channels should be avoided. Even so, cinema commercials and/or YouTube might be the most plausible information channel to avoid; YouTube in particular might backfire and reduce vaccination rates as a result of unsolicited content that undermines county-sanctioned media coverage on vaccines.

Closely examining data from individual cases (counties) corroborate the theory of some conditions backfiring with respect to producing high vaccination uptake. Jonkoping provided vaccination in all schools, but only achieved vaccination uptake among 65% of eligible girls, as opposed to over 80% uptake achieved in the three other counties with vaccination availability at all schools. Notably, Jonkoping used two communication channels (CCY = 1 and SM = 1) that were absent in the other three counties (CCY = 0 and SM = 0).

In sum, the CNA results indicate that whenever vaccination is available at only some schools, media coverage make a difference for high uptake. Furthermore, the common core of the resulting CNA models for HI_UPTAKE = 0 indicate that a lack of media coverage when vaccinations are provided only in some schools make a difference for lower vaccine uptake. By systematically scrutinizing the configurations of implementation strategies associated with high-uptake and low-uptake counties, CNA extends the conclusions drawn from Rehn and colleagues’ regression model.

The data on vaccination uptake in Sweden do not comprise multiple outcomes, and hence, CNA’s capacity to uncover multi-outcome structures cannot be showcased with this example. Readers interested in multi-outcome discovery with CNA are, instead, referred to [39, 44], the first of which finds a causal chain and the second a common-cause structure.

Our approach to setting consistency and coverage thresholds in this study is consistent with the extant CNA literature. Of note, however, is that ensemble strategies have been newly proposed where consistency and coverage thresholds are systematically varied across a series of thresholds in order to measure “fit-robustness,” the degree to which a specific model agrees with other models identified at different consistency and coverage thresholds in the same dataset [42]. While ensemble approaches to setting consistency and coverage thresholds are still nascent, they appear highly promising as analytic strategies that can help mitigate the risk of overfitting models [41].

## Part 3: tips for practitioners

### Recommendations for reporting analyses and results for CCMs

Variation in reporting study design, analysis, and results exists in previously published peer-reviewed CCM literature. To advance the methodological rigor with which CCMs are applied, we offer recommendations for describing design, analysis, and results for CCMs. We also provide additional material in Additional files 1 and 2 accompanying this article to allow for independent replication of these analyses and findings.

We suggest that future studies and publications applying CCMs (1) describe the rationale for using the CCM (e.g., CNA), (2) describe the rationale for selecting which factors (outcomes and conditions) to include in the analysis, (3) describe the process used to assign cases to factor values [45, 46] (e.g., high vaccination uptake), (4) specify the software (and version) used for analysis; (5) describe the iterative analyses used to refine factors (e.g., different approaches to calibration) and models (e.g., adding/dropping factors), (6) list the number of models generated in each iteration of the analysis and identify commonalities across models, (7) report consistency and coverage thresholds for final models along with ranges for models not part of the final model(s), and (8) describe the rationale for selecting the final model(s).

In this article, we used conceptual knowledge of HPV vaccination uptake and the existence of variability across cases to select the factors to include in the initial analysis. Given the structure of the data, we relied on the original binary factor calibration for all but one factor (SCHOOLS), which was calibrated as a multi-value ordinal factor. Moreover, to improve the diversity index, we explored all mutually exclusive combinations of two factors representing information channels into a single factor. We analyzed six datasets using the cna package in R [13]. Five models reached perfect consistency and coverage and, thus, fit the data equally well. All models exhibited a common core, which we reported as determinate causal inference supported by our data. At the same time, we acknowledge that the common core did not amount to a complete causal model. Based on theoretical considerations, which we explicitly described, we then selected one of five viable completions as the most plausible overall model. We reported all relevant model fit scores for our preferred model and provide additional files with detailed descriptions of each step in the analytic process (see Additional file 1), the analytic dataset (see Additional file 2), and the R script (see Additional file 3) to allow for independent replication and verification of our results.

### Limitations of CNA

CNA has several limitations of which implementation researchers should be aware. First, although CNA supports causal inference, there are limitations to the extent to which results may be generalized. Results can be confounded by unmeasured causes that are located on causal paths to outcomes that do not go through any measured factors. If the data cannot be assumed to be homogenous in confounder distributions—meaning that unmeasured factors do not affect all cases/configurations equally—generalization becomes problematic given the risk of over-interpreting the data or incorrectly inferring a causal relationship. As with other CCMs, familiarity with cases helps to evaluate generalizability—by, for example, justifying that cases included in the analysis are homogeneous with respect to potential confounders—and to interpret solutions generated by mathematical modeling. In the example dataset from Rehn and colleagues, it is possible a third factor may explain the negative relationship between cinema commercials and/or YouTube, for example. In this instance, researchers can further explore the relationship between these factors and HPV vaccination uptake through qualitative interviews or other methods to confirm findings.

Second, CCMs rarely uncover the data-generating causal structures in full. Configurational data analyzed in observational studies tend to be fragmented (i.e., exhibit low diversity), so most logically possible combinations of conditions are not present in the observed cases. Under these circumstances, CCMs may reveal only portions of the underlying causal structures. Thus, the fact that some factor *X* is not contained in a CCM model must not be taken to mean that *X* is causally irrelevant. Unless there is reason to assume that the data are non-fragmented, the absence of *X* from a model can only mean that the data do not contain evidence for *X*’s relevance (which is not the same as *X*’s irrelevance).

## Conclusions

CNA offers new insights of potential high interest to implementation researchers. We demonstrated the utility of CNA using data previously analyzed with RAMs. The authors of the original, RAM-based analysis indicated that offering vaccination in schools increased county-level vaccination uptake while no information channels significantly increased vaccination uptake [40]. By contrast, CNA results indicated that under specific conditions, information channels did make a difference for high vaccination uptake. Specifically, our results imply that higher vaccination rates are achieved by either (1) offering the vaccine in all schools or (2) offering the vaccine in some schools and using media coverage but not certain other communication channels.

Compared to RAMs, CCMs have fundamentally different methodological goals and search for different properties of causal structures. CCMs and RAMs answer distinct types of questions. RAMs are useful for estimating the average influence of a specific variable on an outcome while holding other variables constant. CCMs are useful for identifying combinations of specific conditions that may be on the same or different causal paths (i.e., are minimally necessary or sufficient) to an outcome. Of the two CCMs, CNA was built expressly for causal inference and can be used to uncover causal chains underlying the data [13, 14, 39].

CNA has the potential to offer implementation researchers alternative and more nuanced knowledge about causal relationships when examining complex interventions in settings with interdependent or interrelated factors. In particular, CCMs are well-suited for implementation and health services research questions regarding the implementation of multifaceted interventions in complex, real-world settings, the dynamics of which can be influenced by many factors acting in combination.

## Supplementary Information

**Additional file 1.** Includes detailed descriptions of each step performed in the Coincidence Analysis

**Additional file 2.** Provides the full analytic dataset used in this analysis to allow for independent replication and verification of results

**Additional file 3.** Provides the complete R script used in this analysis to support independent replication

## Data Availability

The full dataset used in this analysis along with detailed descriptions of each step of the analysis and the R script are all available for review and replication in additional files accompanying this article (see Additional files 1, 2, and 3). The original dataset is publicly available within the following open-access article: Rehn M, Uhnoo I, Kühlmann-Berenzon S, Wallensten A, Sparén P, Netterlid E. *Highest vaccine uptake after school-based delivery - a county-level evaluation of the implementation strategies for HPV catch-up vaccination in Sweden*. PLoS One. 2016;11(3):e0149857. doi:10.1371/journal.pone.0149857.
