# Correction to: Coincidence analysis: a new method for causal inference in implementation science

**DOI:** 10.1186/s13012-020-01079-8

**Published:** 2021-01-12

**Authors:** Rebecca Garr Whitaker, Nina Sperber, Michael Baumgartner, Alrik Thiem, Deborah Cragun, Laura Damschroder, Edward J. Miech, Alecia Slade, Sarah Birken

**Affiliations:** 1 Duke-Margolis Center for Health Policy, 100 Fuqua Drive, Box 90120, Durham, NC 27708 USA; 2 Duke University School of Medicine, Department of Population Health Sciences, 215 Morris Street, Durham, NC 27701 USA; 3 University of Bergen, Department of Philosophy, Postboks 7805, 5020 Bergen, Norway; 4 University of Lucerne, Frohburgstrasse 3, P.O. Box 4466, 6002 Lucerne, Switzerland; 5 Department of Global Health, College of Public Health, University of South Florida, 3802 Spectrum Boulevard, Tampa, FL 33612 USA; 6 VA Ann Arbor Center for Clinical Management Research, University of Michigan, North Campus Research Complex, 2800 Plymouth Road, Building 18, Ann Arbor, MI 48109-2800 USA; 7 Center for Health Services Research, Regenstrief Institute, 1101 West 10th Street, Indianapolis, IN 46202 USA; 8 Avalere Health, 1201 New York Avenue NW, Suite 1000, Washington, DC 20005 USA; 9 Department of Implementation Science, Wake Forest School of Medicine, 525@Vine Room 5219, Medical Center Boulevard, Winston-Salem, NC 27157 USA

**Correction to: Implement Sci (2020) 15:108**

**https://doi.org/10.1186/s13012-020-01070-3**

Following publication of the original article [1], the authors reported a missing funding declaration in the Acknowledgements declaration. The declaration is the following:

Michael Baumgartner gratefully acknowledges financial support from the Bergen Research Foundation, grant number 811886.

The original article has been updated to include this statement.
