# Supplementary material for: Coincidence analysis: a new method for causal inference in implementation science
Source: Implement Sci. 2020 Dec 11;15:108. doi: 10.1186/s13012-020-01070-3 (PMC7730775; doi:10.1186/s13012-020-01070-3)
Supplement: Supplementary file 1 — Additional file 1. Includes detailed descriptions of each step performed in the Coincidence Analysis [file 13012_2020_1070_MOESM1_ESM.docx]

**Additional file 1. Step-by-Step Guide for Conducting Coincidence Analysis (CNA)**

The document below outlines the steps used to conduct Coincidence Analysis using Rehn and colleagues’ publicly available data on implementation strategies and county-level HPV vaccination uptake in Sweden.(1) The analytic dataset used in the Coincidence Analysis (CNA) is also available in Additional file 2 [see Additional file 2] to allow for independent replication and verification of results.

Research question: What conditions (i.e., delivery settings and information channels) link directly to the outcome (i.e., high catch-up vaccination rates across counties in Sweden)?

| **CNA steps** | **Application of CNA steps in the current study** |
| --- | --- |
| **Step 1: Identify and calibrate values for each factor of interest (i.e., conditions and outcome) to create a dataset for analysis.** | |
| **(a)** Determine, define, and operationalize the outcome of interest. | **(a)** Outcome = county-level catch-up HPV vaccination uptake out of a population of eligible school-aged girls in each county. Vaccination uptake was already determined as the primary outcome of interest in the original study.  Note that with CNA, unlike other Comparative Configurational Methods (CCMs), researchers do not need to distinguish the outcome from the conditions that are hypothesized to lead to that outcome. CNA will automatically identify underlying causal chains in which some conditions lead to an “intermediary outcome” which then leads to the final outcome (assuming such relationships exist in the data).(2) |
| **(b)** Assign and calibrate set membership scores for the outcome. | **(b)** Set membership scores can be assigned using crisp-set (binary), multi-value (categorical), or fuzzy-set (0-1 linear transformation) calibration.(3, 4) Upon reviewing the distribution of the outcome, we assigned a binary score for our outcome variable: high vaccination uptake was defined as counties reporting at least 65% uptake in the eligible population over the study period (1 = high-uptake; 0 = not high-uptake). We conducted sensitivity analyses in which we varied the threshold for “high-uptake,” using two different existing break points in the data. The 65% cut-off generated the greatest diversity among cases for the conditions and the outcome. In a secondary analysis, we modeled the absence of the outcome, or high uptake = 0. Modeling the negation of the outcome can provide additional insights into the phenomenon of study because conditions that prevent the outcome may differ from those that contribute to the presence of the outcome. |
| **(c)** Identify key conditions. | **(c)** The number of conditions relative to the number of cases should be minimized using knowledge of the cases, prior research and theory. This is important because as more conditions are added, the number of logically plausible configurations (combinations of all possible conditions) increases exponentially and limits diversity. We included “schools” as the single vaccination delivery setting in the analysis. The other two delivery settings were not included due to limited variation across counties. In addition, four of the ten information channels were included in the analysis: school-based information, media coverage, social media and YouTube/Cinema commercials. The rationale was that these four implementation strategies exhibited diversity across cases and represented likely communication channels for students and their parents to be informed of why, where, how, and when to access vaccinations. County website and smart phone app were not selected because those strategies require people to actively seek out additional information, so we expected these to reach fewer individuals. |
| **(d)** Assign set membership scores for each condition. | **(d)** The structure of the original dataset guided our set membership scores for the conditions: information channel variables were already dichotomized (1= utilized in the county; 0 = not utilized in the county), while the factor SCHOOLS was assigned a multi-value membership score (0 = no schools, 1 = some schools, 2 = all schools) in which each value was mutually exclusive. |
| **(e)** Create a data matrix of scores for conditions and outcome. | **(e)** A .csv table was created by listing outcome and condition scores for each county (as these are the “cases” we are comparing). See Figure 1 in the main article or the analytic dataset in Additional file 2 [see Additional file 2]. |
| **(f)** Assess diversity of configurations. | **(f)** Determine whether the case configurations are sufficiently diverse to allow for causal inference. With a small to medium number of cases this may be done by reviewing the data matrix. However, with a larger number of cases it is helpful to review the truth table – which can be created using the cna package in R.(2) A truth table lists all of the possible configurations of conditions and outcomes and allows you to see which cases fit within each configuration.  Given that CCMs utilize an iterative approach to analysis, if the data exhibit limited diversity, researchers can explore several options including: modifications to the outcome definition (as we did by considering two different cut points for defining high-uptake), reconsidering which conditions to include in the dataset, re-calibrating the included conditions, or aggregating conditions using the Boolean “and” & “or” operators.  Because we had limited diversity in our data, we decided to disjunctively aggregate two of the information channels—a common CCM approach to reduce the number of conditions without completely eliminating either of the properties represented by the factors from the analysis. Disjunctive aggregation is done by creating a new variable coded as 1 if at least one of two selected conditions is present (e.g., the presence of either condition A “or” condition B); whereas if both are absent the new variable is coded 0. Although not used in our study, conjunctive aggregation is also possible. In forming a conjunct, the new variable would be coded as 1 if both conditions are present (the presence of condition A “and” condition B); whereas if only one condition or neither conditions are present then it is coded 0. |
| **Step 2: Perform CNA using the cna package in R.**[2] **[Note: R is the only statistical program that can currently be used to conduct CNA]** | |
| **(a)** Set minimum consistency and coverage scores for identifying minimally sufficient and minimally necessary conditions. | **(a)** Consistency is the extent to which a particular condition or whole causal model is associated with a given outcome. Coverage is the extent to which a given outcome is associated with a particular condition or whole causal model.(5) The CNA algorithm searches for conditions and combinations of conditions in the data that meet the default consistency and coverage thresholds which are both 1.0 in the cna software package for R. Because we found solutions at perfect consistency and coverage, we did not have to lower consistency and coverage thresholds below 1.0. In principle, researchers can lower the thresholds if there are no solutions with perfect consistency and coverage.  Significantly lowering consistency and coverage thresholds increases the risk of over-interpreting the data and incorrectly inferring a causal relationship. Lower consistency values may indicate lower confidence in the causal relationship between conditions and the outcome. Low coverage for a solution set may indicate that there are confounding factors not included in the model. [NOTE: Ensemble strategies have been newly proposed where consistency and coverage thresholds are systematically varied across a series of thresholds in order to measure “fit-robustness,” the degree to which a specific model agrees with other models identified at different consistency and coverage thresholds in the same dataset. (6)While ensemble approaches to setting consistency and coverage thresholds are still nascent, they appear highly promising as analytic strategies that can help mitigate the risk of overfitting models (7), and implementation researchers should strongly consider this alternative approach to setting consistency and coverage thresholds.] |
| **(b**) Identify minimally sufficient and minimally necessary conditions. | **(b)** In CNA, all sufficient conditions built from the bottom up are automatically redundancy-free, i.e. minimally sufficient conditions (msc). Our primary analysis identified implementation strategies – the conditions and combinations of conditions – associated with high-uptake counties (HI_UPTAKE=1). A secondary analysis identified implementation strategies associated with low-uptake counties (HI_UPTAKE=0). |
| **Step 3: Interpret results and refine model inputs if necessary by either recalibrating outcomes or conditions, or modifying which conditions are included in the analysis.** | |
|  | Atomic solution formulas (asf) consist of minimally necessary disjunctions of minimally sufficient conditions for a given outcome. If CNA uncovers intermediary outcomes, the asf are combined into a single solution called complex solution formulas (csf) which are indicative of causal chains, none of which were uncovered in the current study.  As mentioned previously, as part of the iterative approach to analysis, researchers can conduct additional sensitivity analyses in which the outcomes or conditions can be recalibrated, or the conditions included in the analysis can be modified. |
|  | Results of our primary analysis were ambiguous which means that the data substantiated more than one possible solution. While our analysis yielded ambiguous results, the findings did suggest the following minimally sufficient disjunction of minimally sufficient conditions was at least part of the overall solution:  $\mathrm{SCHOOLS}\text{=2} + SCHOOLS\text{=1}*MC\text{=1}$  When CNA produces multiple solution formulas indicating ambiguity in the model results, this does not mean that the results are uninformative. Our results implied that the availability of vaccination in some schools (SCHOOLS=1) was only sufficient for high vaccination rates if media coverage (MC=1) was employed and certain other communication channels were not utilized. While the data contained enough evidence to infer that when vaccination is available at some but not all schools, availability must be complemented by media coverage to reach high uptake, the data did not contain enough evidence to determine exactly which other communication channels were to be avoided. Parsing through solutions in conjunction with theories, frameworks, and details about the cases can help hypothesize which of the solution models may be favored and help in considering how and why the conditions might lead to the outcome. |

**REFERENCE LIST**

1. Rehn M, Uhnoo I, Kuhlmann-Berenzon S, Wallensten A, Sparen P, Netterlid E. Highest Vaccine Uptake after School-Based Delivery - A County-Level Evaluation of the Implementation Strategies for HPV Catch-Up Vaccination in Sweden. Plos One. 2016;11(3).

2. Ambühl M, Baumgartner M, Epple R, Thiem A. cna: Causal Modeling with Coincidence Analysis. 2020.

3. Ragin CC. Qualitative Comparative Analysis Using Fuzzy Sets (fsQCA). In: Rihoux B, Ragin CC, editors. Configurational Comparative Methods. Thousand Oaks, CA: SAGE Publications, Inc; 2009. p. 87-121.

4. Skaaning SE. Assessing the Robustness of Crisp-set and Fuzzy-set QCA Results. Sociological Methods & Research. 2011;40(2):391-408.

5. Ragin CC. Set relations in social research: Evaluating their consistency and coverage. Polit Anal. 2006;14(3):291-310.

6. Parkkinen V, Baumgartner M. Robustness and Model Selection in Configurational Causal Modeling. Sociological Methods & Research. 2020.

7. Arel-Bundock V. The Double Bind of Qualitative Comparative Analysis. Sociological Methods & Research. 2019:0049124119882460.
