# Supplementary material for: Coincidence analysis: a new method for causal inference in implementation science
Source: Implement Sci. 2020 Dec 11;15:108. doi: 10.1186/s13012-020-01070-3 (PMC7730775; doi:10.1186/s13012-020-01070-3)
Supplement: Supplementary file 2 — Additional file 2. Provides the full analytic dataset used in this analysis to allow for independent replication and verification of results [file 13012_2020_1070_MOESM2_ESM.docx]

Additional file 2.

The analytic dataset that was used to conduct the Coincidence Analysis in the main article is provided below to allow for independent replication and verification of results.

|  | HI_UPTAKE | OUTCOME | SCHOOLS | AS | SS | MC | CCY | TI | SP | CW | SBI | LI | AD | SM | OHC | HC | PHC | |
| --- | --- | --- | --- | --- | --- | --- | --- | --- | --- | --- | --- | --- | --- | --- | --- | --- | --- | --- |
| Västernorrland | 1 | 0.84 | 2 | 1 | 0 | 0 | 0 | 0 | 0 | 0 | 1 | 0 | 0 | 0 | 0 | 0 | 1 | |
| Jämtland | 1 | 0.82 | 2 | 1 | 0 | 0 | 0 | 0 | 0 | 1 | 1 | 0 | 1 | 0 | 0 | 0 | 1 | |
| Värmland | 1 | 0.82 | 2 | 1 | 0 | 0 | 0 | 0 | 0 | 0 | 1 | 1 | 0 | 0 | 0 | 0 | 1 | |
| Jönköping | 1 | 0.65 | 2 | 1 | 0 | 0 | 1 | 0 | 0 | 1 | 1 | 1 | 1 | 1 | 0 | 0 | 1 | |
| Öbrero | 1 | 0.75 | 1 | 0 | 1 | 1 | 0 | 0 | 0 | 0 | 1 | 1 | 1 | 0 | 0 | 0 | 1 | |
| Dalarna | 1 | 0.68 | 1 | 0 | 1 | 1 | 0 | 0 | 0 | 0 | 0 | 1 | 1 | 0 | 0 | 0 | 1 | |
| Halland | 1 | 0.68 | 1 | 0 | 1 | 1 | 0 | 0 | 0 | 1 | 0 | 1 | 1 | 1 | 1 | 0 | 1 | |
| Västerbotten | 0 | 0.60 | 1 | 0 | 1 | 0 | 1 | 1 | 0 | 1 | 0 | 1 | 1 | 0 | 0 | 0 | 1 | |
| Skåne | 0 | 0.59 | 1 | 0 | 1 | 1 | 1 | 0 | 0 | 1 | 1 | 1 | 1 | 1 | 1 | 1 | 1 | |
| Västmanland | 0 | 0.55 | 1 | 0 | 1 | 0 | 1 | 0 | 0 | 0 | 1 | 1 | 1 | 0 | 0 | 0 | 1 | |
| Stockholm | 0 | 0.54 | 1 | 0 | 1 | 0 | 0 | 0 | 0 | 0 | 1 | 1 | 1 | 1 | 0 | 0 | 1 | |
| Västra Götaland | 0 | 0.52 | 1 | 0 | 1 | 0 | 1 | 1 | 0 | 0 | 0 | 1 | 1 | 1 | 0 | 0 | 1 | |
| Östergötland | 0 | 0.60 | 0 | 0 | 0 | 0 | 1 | 0 | 1 | 1 | 0 | 1 | 1 | 0 | 0 | 0 | 1 | |
| Kalmar | 0 | 0.59 | 0 | 0 | 0 | 0 | 0 | 0 | 0 | 0 | 0 | 1 | 0 | 0 | 0 | 0 | 1 | |
| Norrbotten | 0 | 0.58 | 0 | 0 | 0 | 1 | 0 | 0 | 0 | 0 | 1 | 1 | 1 | 1 | 0 | 0 | 1 | |
| Gävleborg | 0 | 0.55 | 0 | 0 | 0 | 1 | 0 | 0 | 0 | 1 | 0 | 1 | 1 | 0 | 0 | 0 | 1 | |
| Blekinge | 0 | 0.53 | 0 | 0 | 0 | 0 | 0 | 0 | 0 | 0 | 0 | 1 | 1 | 0 | 0 | 0 | 1 | |
| Kronoberg | 0 | 0.53 | 0 | 0 | 0 | 1 | 0 | 0 | 0 | 0 | 0 | 1 | 0 | 0 | 0 | 0 | 1 | |
| Södermanland | 0 | 0.53 | 0 | 0 | 0 | 0 | 1 | 0 | 1 | 1 | 1 | 1 | 1 | 0 | 0 | 0 | 1 | |
| Gotland | 0 | 0.51 | 0 | 0 | 0 | 0 | 0 | 0 | 0 | 0 | 0 | 1 | 1 | 0 | 0 | 0 | 1 | |
| Uppsala | 0 | 0.49 | 0 | 0 | 0 | 1 | 1 | 0 | 1 | 1 | 0 | 1 | 1 | 0 | 0 | 1 | 1 | |
| *HI_UPTAKE = vaccination uptake >= 65%; OUTCOME = Raw vaccination uptake rate; SCHOOLS = all schools (2) some schools (1) no schools (0); AS = All schools; SS = Some schools ; MC = media coverage; CCY = Cinema commercial/You Tube; TI = Targeted Information; SP = Smart phone app; CW = county website; SBI = School-based information; LI = Letter/invitation; AD = Advertisement; SM = Social media; OHC = Online health care consulting; HC = Health care center; PHC = Primary health care center | | | | | | | | | | | | | | | | | |  |

SOURCE FOR ORIGINAL DATASET: Rehn M, Uhnoo I, Kühlmann-Berenzon S, Wallensten A, Sparén P, Netterlid E. Highest vaccine uptake after school-based delivery - a county-level evaluation of the implementation strategies for HPV catch-up vaccination in Sweden. PLoS One. 2016;11:e0149857. doi:10.1371/journal.pone.0149857.
