# Supplementary material for: Coincidence analysis: a new method for causal inference in implementation science
Source: Implement Sci. 2020 Dec 11;15:108. doi: 10.1186/s13012-020-01070-3 (PMC7730775; doi:10.1186/s13012-020-01070-3)
Supplement: Supplementary file 3 — Additional file 3. Provides the complete R script used in this analysis to support independent replication [file 13012_2020_1070_MOESM3_ESM.docx]

Additional file 3. R script used in Coincidence Analysis is provided below for purposes of independent replication.

library(cna)

# Data with ternary SCHOOLS variable and HI_UPTAKE, calibrated at 65%:

HK <- structure(list(HI_UPTAKE = c(1L, 1L, 1L, 1L, 1L, 1L, 1L, 0L,

0L, 0L, 0L, 0L, 0L, 0L, 0L, 0L, 0L, 0L, 0L, 0L, 0L), TI = c(0L,

0L, 0L, 0L, 0L, 0L, 0L, 1L, 0L, 0L, 0L, 1L, 0L, 0L, 0L, 0L, 0L,

0L, 0L, 0L, 0L), SP = c(0L, 0L, 0L, 0L, 0L, 0L, 0L, 0L, 0L, 0L,

0L, 0L, 1L, 0L, 0L, 0L, 0L, 0L, 1L, 0L, 1L), CW = c(0L, 1L, 0L,

1L, 0L, 0L, 1L, 1L, 1L, 0L, 0L, 0L, 1L, 0L, 0L, 1L, 0L, 0L, 1L,

0L, 1L), MC = c(0L, 0L, 0L, 0L, 1L, 1L, 1L, 0L, 1L, 0L, 0L, 0L,

0L, 0L, 1L, 1L, 0L, 1L, 0L, 0L, 1L), SBI = c(1L, 1L, 1L, 1L,

1L, 0L, 0L, 0L, 1L, 1L, 1L, 0L, 0L, 0L, 1L, 0L, 0L, 0L, 1L, 0L,

0L), LI = c(0L, 0L, 1L, 1L, 1L, 1L, 1L, 1L, 1L, 1L, 1L, 1L, 1L,

1L, 1L, 1L, 1L, 1L, 1L, 1L, 1L), AD = c(0L, 1L, 0L, 1L, 1L, 1L,

1L, 1L, 1L, 1L, 1L, 1L, 1L, 0L, 1L, 1L, 1L, 0L, 1L, 1L, 1L),

SM = c(0L, 0L, 0L, 1L, 0L, 0L, 1L, 0L, 1L, 0L, 1L, 1L, 0L,

0L, 1L, 0L, 0L, 0L, 0L, 0L, 0L), CCY = c(0L, 0L, 0L, 1L,

0L, 0L, 0L, 1L, 1L, 1L, 0L, 1L, 1L, 0L, 0L, 0L, 0L, 0L, 1L,

0L, 1L), OHC = c(0L, 0L, 0L, 0L, 0L, 0L, 1L, 0L, 1L, 0L,

0L, 0L, 0L, 0L, 0L, 0L, 0L, 0L, 0L, 0L, 0L), OHC.1 = c(0L,

0L, 0L, 0L, 0L, 0L, 0L, 0L, 1L, 0L, 0L, 0L, 0L, 0L, 0L, 0L,

0L, 0L, 0L, 0L, 1L), SCHOOLS = c(2L, 2L, 2L, 2L, 1L, 1L,

1L, 1L, 1L, 1L, 1L, 1L, 0L, 0L, 0L, 0L, 0L, 0L, 0L, 0L, 0L

)), class = "data.frame", row.names = c("Vaesternorrland",

"Jaemtland", "Vaermland", "Joenkoeping", "Oerebro", "Dalarna",

"Halland", "Vaesterbotten", "Skane", "Vaestmanland", "Stockholm",

"Vaestra Goetaland", "Oestergoetland", "Kalmar", "Norrbotten",

"Gaevleborg", "Blekinge", "Kronoberg", "Soedermanland", "Gotland",

"Uppsala"))

# Theoretically selected variables:

# -----------------------------

# school-based info SBI

# media coverage MC

# social media SM

# YouTube/cinema/commercial CCY

# + SCHOOLS

# Data resulting from theoretical factor selection

dat <- HK[,c("HI_UPTAKE","SCHOOLS", "SBI", "MC", "CCY", "SM")]

# Aggregate two information channels disjunctively:

# Go through all possibilities

agg_fac <- combn(c("SBI", "MC", "CCY", "SM"), 2)

sol.list <- vector("list", ncol(agg_fac))

# Analysis LOOP

# -------------

for (i in 1:ncol(agg_fac)){

cat(i, "\n")

x <- dat2

y <- ifelse((x[,which(names(x) %in% agg_fac[1,i])]==1) | (x[,which(names(x) %in% agg_fac[2,i])]==1), 1, 0)

z <- cbind(x,y)

colnames(z)[colnames(z)=="y"] <- paste0(agg_fac[1,i],"_",agg_fac[2,i])

z_ana <- z[,-which(names(z) %in% c(agg_fac[1,i],agg_fac[2,i]))]

result <-subset(asf(mvcna(z_ana, ordering=list("HI_UPTAKE"), strict = T, con=1,cov=1,maxstep=c(4,4,14))), outcome=="HI_UPTAKE=1")

sol.list[[i]] <- list(result,z_ana)

}

# List of solutions with corresponding datasets

sol.list

# all solutions / causal models

do.call(rbind,lapply(sol.list, `[[`, 1))

# Common core, expression (1)

print(mvcond("SCHOOLS=2 + SCHOOLS=1*MC=1 <-> HI_UPTAKE=1", dat),show.cases=T)
